# Structural Features of Carbons Produced Using Glucose, Lactose, and Saccharose

**DOI:** 10.1186/s11671-016-1723-z

**Published:** 2016-11-17

**Authors:** Ivan F. Myronyuk, Volodymyr I. Mandzyuk, Volodymyr M. Sachko, Volodymyr M. Gun’ko

**Affiliations:** 1 Vasyl Stefanyk Precarpathian National University, 57 Shevchenko Street, 76018 Ivano-Frankivsk, Ukraine; 2 Chuiko Institute of Surface Chemistry, 17 General Naumov Street, 03164 Kyiv, Ukraine

**Keywords:** Activated carbons, Thermal degradation processes, Porous structure, surface functional groups, Specific conductivity, 81.05.Rm, 81.05.Uw, 87.14.Df

## Abstract

Glucose, lactose, and saccharose were used as precursors to prepare chars at 400 °C then activated at 800 °C or 1000 °C in closed vessels with controlled amounts of oxygen penetrating through nanopores in the vessel walls. There are correlations between the porosity, amounts of residual O- and H-containing functionalities, and electroconductivity of amorphous carbons studied. The pore size distributions calculated using the nitrogen adsorption isotherms and TEM images show that all carbons are mainly nanoporous with certain contribution of narrow mesopores (at pore half-width *x* < 5 nm). Oxidizing activation by oxygen penetrating into the closed vessels with chars through nanopores can more strongly change the outer layers of char particles than the inner pores. Therefore, despite relatively great burn-off degree, the textural characteristics are relatively low for activated carbons.

**Electronic supplementary material:**

The online version of this article (doi:10.1186/s11671-016-1723-z) contains supplementary material, which is available to authorized users.

## Background

Carbons are frequently produced using natural row materials, which include carbohydrates [1–13]. Carbohydrates (e.g., saccharides) are appropriate materials for carbonization because they include many well-removed O/H-containing functionalities [14–25]. Therefore, their carbonization with the dehydration as one of the main processes can be carried out at temperatures (320–500 °C) lower than that used for carbonization of other compounds, e.g., phenolformaldehyde resin at 800 °C [26–28]. To improve the textural characteristics of chars, they are activated in the atmosphere with water vapor, CO_2_, or CO at 800–1100 °C [1–3, 29]. Carbonization of precursors mixed with such active compounds as zinc chloride, phosphorus acid, and potassium carbonate can provide a high porosity of the carbons without of additional activation [30–32].

The physicochemical properties of the carbonaceous materials depend strongly on the characteristics of their precursors. Since chars are composed of polyaromatic structures (graphenes), the presence of cyclic fragments in the precursors is favorable for effective carbonization. Therefore, thermolysis of mono- or disaccharides results in the formation of polyaromatic compounds, whose condensation gives graphene clusters. Among simple carbohydrates, saccharose is frequently used as a char precursor [13–25, 33–35]. Mono- and disaccharides are characterized by tautomerism and diastereoisomerism, e.g., besides chain-cyclic tautomerism of glucose, there are eight pairs of its diastereomers. The tautomers and diastereomers (stereoisomers not related as mirror images) are characterized by different physicochemical properties [36, 37]. This can affect the carbonization results for saccharides. Note that carbonization of glucose or other small organic molecules at a surface of oxide materials (such as silica gels, fumed silica, titania, and mixed oxides) typically results in the formation of nonporous carbon nanoparticles similar to those of carbon black [38, 39]. Besides dependences of the textural characteristics of chars on the structure of different saccharides used as precursors, there is an additional question on the effects of activation of chars by small controlled portions of oxygen. Therefore, the aim of this work was to study the thermolysis of glucose (as a monosaccharide) and two disaccharides (such as lactose composed of β-d-galactopyranose and α-d-glucopyranose and saccharose composed of α-d-glucopyranose and β-d-fructofuranose), and the characteristics of the chars (carbonized at 400 °C) and related carbons activated at 800 and 1000 °C using controlled amounts of oxygen.

## Methods

Crystalline monohydrates of glucose, lactose, and anhydrous saccharose were used as precursors of chars prepared at 400 °C for 30 min in air. Oxidizing activation of chars was carried out in ceramic crucibles at 800 or 1000 °C for 30 min. Oxygen (from air) penetrated into the crucibles through mesoporous walls (porosity ~20%). The pore sizes in the walls were decreased by fourfold to fivefold impregnation with a concentrated solution of AlONO_3_ 2H_2_O, dried, and then calcined at 800 °C. Mesoporous alumina filling macropores of the crucible walls has pores of approximately 5 nm in size. Caps of the crucibles were sealed using asbestos strings.

Thermal degradation of glucose, lactose, and saccharose in 25–1000 °C range was studied using thermogravimetry (TG) with a STA 449 F3 Jupiter (Netzsch) apparatus at a heating rate of 5 °C*/*min in the inert (argon) atmosphere.

The textural characteristics of chars and activated carbons, AC (degassed at 180 °C for 24 h), have been determined using the nitrogen adsorption-desorption isotherms recorded at 77.4 K using a Quantachrome Autosorb Nova 2200e adsorption analyzer. The pore size distributions (PSDs) (differential ~ d*V*/d*x*) have been calculated using the quenched solid density functional theory (QSDFT, Quantachrome software) [40] and 2D-nonlocal DFT (2D-NLDFT, SAIEUS program, Micromeritics software) [41, 42] methods with the slit-shaped pore model. Additionally, the complex model with slit-shaped (S) and cylindrical (C) pores and voids (V) between nanoparticles (SCV-model) with self-consisting regularization (SCR) procedure was used [43]. The differential PSD functions with respect to the pore volume (PSD_V_, *f*_V_(*x*) ~ *dV*/*dx*, ∫*f*_V_(*x*)*dx* ~ *V*_p_) and the specific surface area (PSD_S_, *f*_S_(*x*) ~ *dS*/*dx*, ∫*f*_S_(*x*)*dx* ~ *S*) were used to estimate the contribution of nano- (*x* < 1 nm), meso- (1 < *x* < 25 nm), and macropores (*x* > 25 nm).

The X-ray diffraction (XRD) patterns of samples treated at different temperatures were recorded at room temperature using a DRON-4-07 (Burevestnik, St. Petersburg) diffractometer with Cu K_α_ (*λ* = 0*.*154178 nm) radiation and a Ni filter in the 2θ range from 10° to 65° with a step of 0.1° using the Bragg-Brentano geometry.

Infrared (IR) spectra were recorded using a Specord M80 spectrometer over the 400–4000 cm^−1^ range. A sample (4 mg) was stirred with dry KBr (weight ratio 1:100), treated in a stainless steel microbreaker for 10 min, and pressed in a thin transparent plate of 20 × 5 mm^2^ in size.

Transmission electron microscopy (TEM) images have been obtained using a JEOL JSM-2100F field emission TEM operated at 200 kV. TEM images have been analyzed using Fiji (local thickness plugin) [44] and ImageJ (granulometry plugin) [45, 46] software.

Desorption of fragments with C_4_, C_5_, C_6_, and C_9_, as well as CO, CO_2_, and others, was analyzed using temperature-programmed desorption (TPD) with mass-spectroscopic (MS) control using a MX 7304A mass spectrometer (Electron, Sumy, Ukraine, sensitivity ~10^−8^ g at m/z 1–400) [47, 48]. A sample (∼4 mg) placed in a quartz–molybdenum cell (diameter 11 mm) was degassed at room temperature and pressure 7 × 10^−5^ Pa and then heated to 800 °C at a linear heating rate of 1 °C*/*min. Volatile products are desorbed from the sample, ionized by the electron beam, separated by a mass-analyzer, and brought to a detector.

Impedance analysis of carbon samples was carried out using a Metrohm Autolab FRA-2 (Frequency Response Analyzer) at 10^−2^–10^5^ Hz and voltage amplitude of 10 mV [49].

## Results and Discussion

**Fig. 1:**
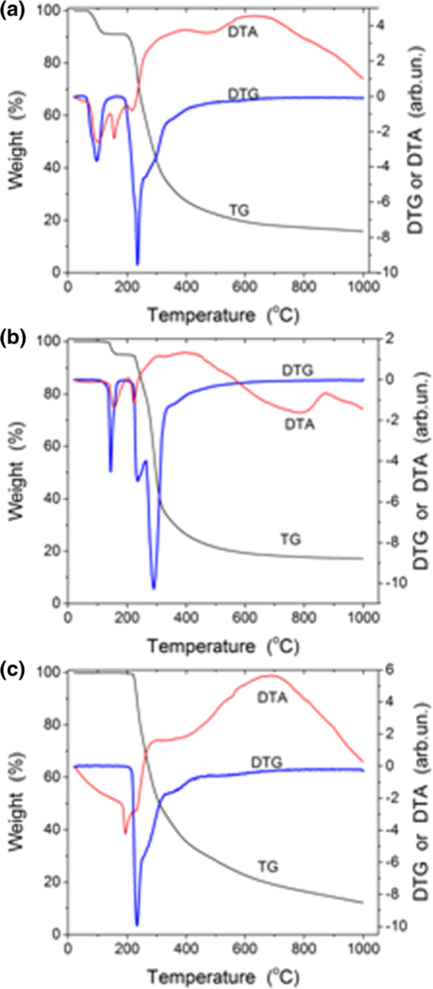
Saccharide thermolysis corresponding to mass loss (thermogravimetry (*TG*)), differential TG (*DTG*), and differential thermal analysis (*DTA*) curves for **a** glucose, **b** lactose, and **c** saccharose

The thermolysis of glucose, lactose, and saccharose in the inert atmosphere (Fig. 1, Table 1) shows several processes: desorption of intact water (65–145 °C for glucose and 100–175 °C for lactose as crystalline hydrates), melting, caramelization, dehydration (associative desorption of water molecules), carbonization (formation of polyaromatics and carbon sheets and graphenes), and structure transformation of chars.

**Table 1:** Processes observed during heating of precursors to 1000 °C

Processes	Glucose	Saccharose	Lactose
	C_6_H_12_O_6_·H_2_O	C_12_H_22_O_12_	C_12_H_22_O_11_·H_2_O
Intact water removal			
Temperature (°C)	65–145	–	100–175
Water loss (mole)	1	–	1
Melting			
Temperature (°C)	160	197	220
Dehydration			
Temperature (°C)	200–230	218–260	220–230
Water loss (mole)	1	5	3
Carbonization			
Temperature (°C)	230–332	260–355	230–343
Water loss (mole)	5	6	8
Residual mass (%)	40	42	40
Char structure changes			
Temperature (°C)	332–1000	355–1000	343–1000
Residual mass (%)	16	12	17
Mass loss in this process (%)	60	75	55

The maximal rate of removal of intact water is at 96 °C for glucose and 144 °C for lactose (Fig. 1a, b, DTG). The processes of water removal, caramelization (~160 °C), melting (160, 220, and 197 °C for glucose, lactose, and saccharose, respectively), and dehydration are endothermic ones (Fig. 1, DTA minima). Removal of water due to the condensation reactions between the hydroxyl groups of interacting molecules and the formation of polyaromatic compounds occur at 200–330 °C (glucose), 230–355 °C (lactose), and 218–345 °C (saccharose). These processes result in the mass loss (Table 1). The residual mass corresponds to 40% (glucose and lactose) and 42% (saccharose) with respect to the initial precursor mass. This corresponds to the loss of a major fraction of structural water due to condensation of O-containing functionalities. Subsequent heating to 1000 °C leads to the mass loss up to 88% (saccharose), 84.3% (glucose), and 82.9% (lactose). Residual carbon remains because the process was carried out in the inert atmosphere.

**Fig. 2:**
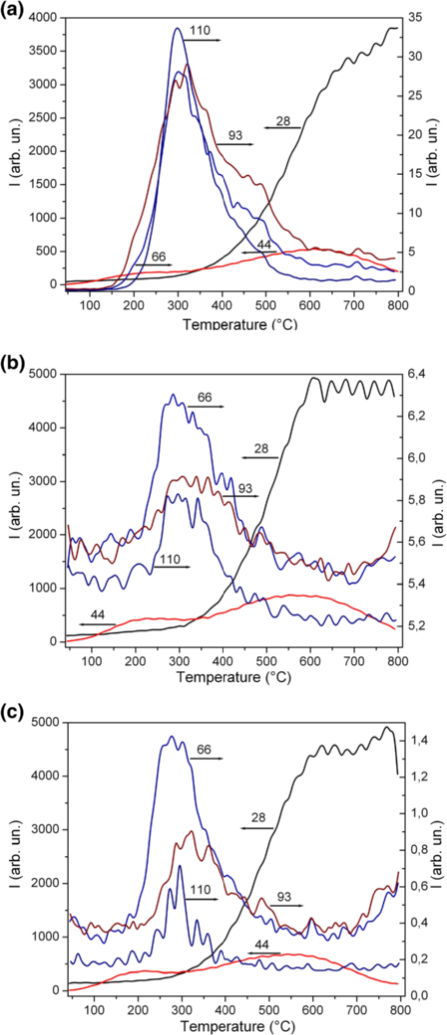
TPD mass-spectra of chars **a** G400, **b** L400, and **c** S400 with lines of fragments C_4_H_2_O (m/z 66), C_5_HO_2_ or C_6_H_5_O (m/z 93), C_6_H_6_O_2_ or C_9_H_2_ (m/z 110), and molecules CO_2_ (m/z 44) and CO (m/z 28)

**Fig. 3:**
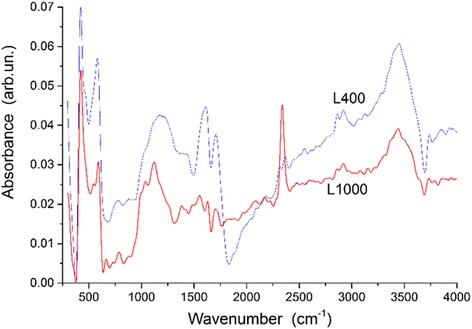
IR spectra of samples L400 and L1000

The most intensive lines in the TPDMS thermograms of chars correspond to the elimination of CO and CO_2_ (Fig. 2). This suggests that the residual amounts of O-containing functionalities in the chars are relatively great. Certain amounts of H-containing functionalities should be remained in the chars. These results are in agreement with the IR spectra of L400 (Fig. 3) showing the presence both C–H (bands at 2924–2916 and 2868 cm^−1^) and O–H (a broad band at 3450 cm^−1^) functionalities in the chars. The maximal amounts of desorbed fragments (such as C_4_H_2_O at m/z 66, C_5_HO_2_ or C_6_H_5_O at m/z 93, and C_6_H_6_O_2_ or C_9_H_2_ at m/z 110) are observed at ~300 °C (mainly in the range of 200–500 °C, Fig. 2). However, CO elimination is maximal at 600–800 °C that corresponds to structural changes in the chars observed in the TG/DTG/DTA thermograms at high temperatures (Fig. 1).

**Fig. 4:**
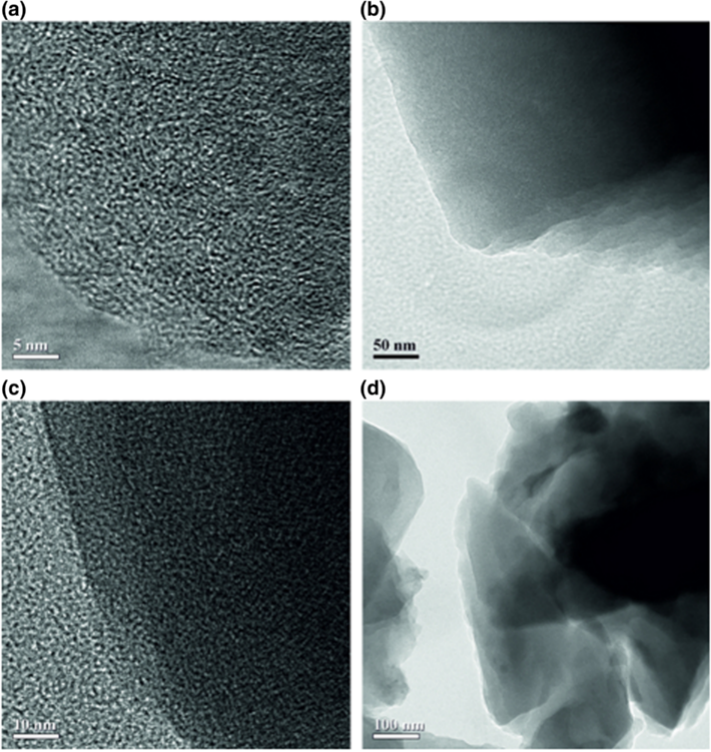
TEM images of carbons (**a**, **b**) L800 (*scale bar* 5 and 50 nm) and (**c**, **d**) S800 (*scale bar* 10 and 100 nm)

**Fig. 5:**
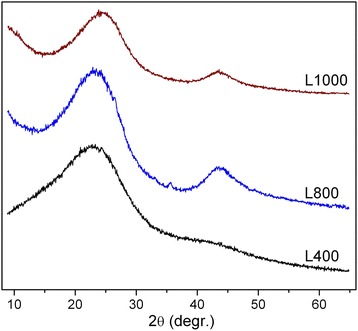
XRD patterns of L400, L800, and L1000

Carbonization of a melted precursor is accompanied by elimination of water vapor that results in boiling up of the reacted precursor. This provides the formation of pores in the chars. The chars and AC have a lamellar shape (Fig. 4b, d) with a stack thickness of 5–40 nm. Nanosized graphenes (1–5 nm in size) form a random amorphous structure of the carbons (Fig. 4a, c). The XRD patterns of lactose-based char and carbons (Fig. 5) and electron diffraction patterns (see Additional file 1: Figure S1) confirm that the materials are amorphous.

**Fig. 6:**
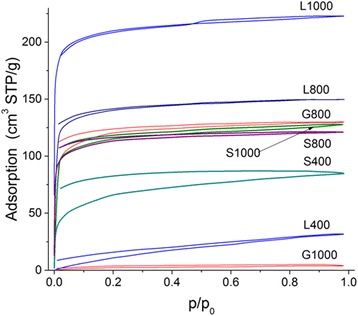
Nitrogen adsorption-desorption isotherms for chars (L400, S400) and AC

**Fig. 7:**
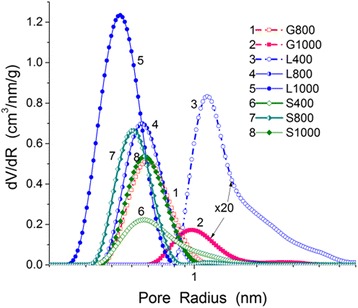
Pore size distributions calculated using the 2D-NLDFT method with the slit-shaped pore model

**Fig. 8:**
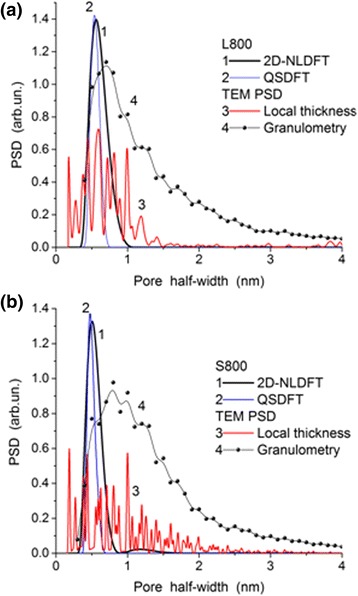
Pore size distributions of L800 (**a**) and S800 (**b**) calculated using the 2D-NLDFT and QSDFT methods with the slit-shaped pore model and TEM images (Fig. 6a, c) treated using Fiji (local thickness plugin) and ImageJ (granulometry plugin) software

The most nitrogen adsorption-desorption isotherms of the carbons studied are characterized by open hysteresis loops (Fig. 6). This can be due to the presence of long and narrow pores with narrow necks close in size to the nitrogen molecules [50]. As a whole, all samples are nanoporous with a certain contribution of narrow mesopores (Table 2, Figs. 7 and 8, and in Additional file 1: Figure S2). Note that the G400 sample was with very low open porosity; therefore, the nitrogen adsorption isotherm was not recorded. However, this sample has certain closed porosity because the specific surface area estimated from the SAXS data corresponds to 185 m^2^/g (the procedure of SAXS investigation is described in detail in [51]).

**Table 2:** Textural characteristics of chars and activated carbons

Sample	Burn-off (%)	*ρ*_b_ (g/cm^3^)	*S*_BET_ (m^2^/g)	*V*_p_ (cm^3^/g)	*S*_nano_ (m^2^/g)	*S*_meso_ (m^2^/g)	*V*_nano_ (cm^3^/g)	*V*_meso_ (cm^3^/g)	*S*_QSDFT_ (m^2^/g)	*S*_NLDFT_ (m^2^/g)
G400	42^a^	0.29	185^c^							
G800	22^b^	0.64	383	0.201	181	202	0.086	0.113	334	263
G1000	56^b^	0.60	10	0.006	2	8	0.001	0.005	8	7
L400	42^a^	0.50	62	0.049	2	60	0.001	0.046	39	31
L800	32^b^	0.54	437	0.232	318	119	0.162	0.070	395	318
L1000	46^b^	0.64	652	0.345	497	155	0.226	0.118	714	626
S400	44^a^	0.50	223	0.131	148	75	0.069	0.061	211	166
S800	28^b^	0.77	356	0.187	204	152	0.092	0.095	353	280
S1000	42^b^	0.75	362	0.198	225	137	0.115	0.083	316	241

With respect to the weight of a ^a^precursor or a ^b^char, *ρ*_b_ is the bulk density, ^c^the specific surface area was calculated using the SAXS data, and the values of *S*_nano_, *V*_nano_, *S*_meso_, and *V*_meso_ were calculated using the SCV/SCR method

An increase in the activation temperature from 800 to 1000 °C for lactose- and saccharose-based chars leads to the enhancement of the porosity (Table 2, *V*_p_, *S*_BET_). However, direction of changes in the PSD is opposite for these samples with increasing temperature (Fig. 7), and the effect is much smaller for saccharose-based carbons.

Note that the SCV/SCR method gives slightly broader PSD (see Additional file 1: Figure S2) than the 2D-NLDFT or QSDFT methods with the slit-shaped pore model (Figs. 7 and 8). However, the PSDs based on the TEM images (Fig. 8) show the presence of narrow mesopores similar to that of the SCV/SCR PSD. Therefore, the textural characteristics shown in Table 2 are based on the SCV/SCR results.

The XRD patterns are shown only for lactose-based carbons (Fig. 5) as representative materials (other carbons have similar XRD patterns). For L400, there is the main line at 2θ = 22° and a weak features at 43°. The second XRD reflection is much intensive for L800 (23° and 43°) and L1000 (24.2° and 43.6°). The inter-plate distances estimated from these values correspond to 0.404 and 0.199 nm (L800) and 0.367 and 0.207 nm (L1000). For graphite, these distances correspond to 0.338 and 0.202 nm [1–3]. Thus, increasing in the activation temperature leads to certain ordering of the carbon structure; however, it remains turbostratic [1–3] with increased inter-plate distances in comparison with those in graphite.

The IR spectra of lactose-based carbons (as representatives) show (Fig. 3) certain changes in the composition of the material during partial oxidizing (activation) at 1000 °C. For L400, the bands at 579, 1490, and 1600 cm^−1^ correspond to different vibrations of the CC bonds in the aromatic rings [52–54]. For L1000, these bands shift to 594, 1555, and 1632 cm^−1^ that correspond to certain ordering of the structure and a decrease in the CC bond length. Some bands (1388, 667, 2349 cm^−1^) correspond to vibrations in CO_2_ [52–56] bound to the carbons. For L1000, an intensive band *ν*_3_ = 2343 cm^−1^ and less intensive band *ν*_1_ = 1381 cm^−1^ shift toward lower wavenumbers in contrast to the band *ν*_2_ = 672 cm^−1^. This corresponds certain changes in the surroundings of the mentioned structures during oxidizing activation with increasing temperature. The bands at 1712 and 782 cm^−1^ (L400) and 1703 and 790 cm^−1^ (L1000) correspond to the vibrations of the >C=O bonds (C is located in the aromatic ring). A broad band at 1121–1184 cm^−1^ corresponds to the C–O stretching vibrations [53, 54]. As mentioned above, there are the bands corresponding to the O–H (~3450 cm^−1^) and C–H (2924–2916 and 2868 cm^−1^) stretching vibrations of the functionalities bound to carbons. Some of them can be located in closed pores or in places poorly accessible for oxygen (similar to nitrogen molecules, Fig. 6). Therefore, they were not removed during oxidizing activation.

**Fig. 9:**
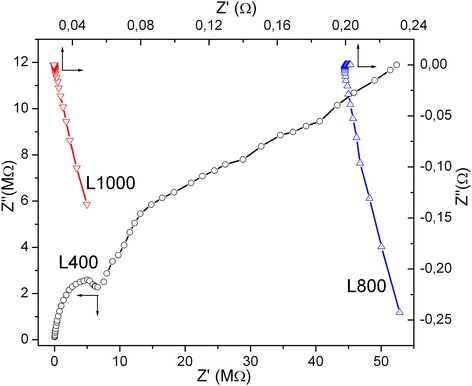
Nyquist plots for lactose-based char (L400) and activated carbons (L800 and L1000)

Char L400 is practically nonconducting (Fig. 9) because the values of both real (*Z*′) and imaginary (*Z*′′) components of the impedance (Z*) are high. The main component of the value of *Z*′′ is the capacitive reactance caused by the barrier capacity at the boundary of carbon particles and the capacity of particles per se [57]. The main reason of this result for L400 is incomplete carbonization to form pure carbon matter. Oxidizing activation of the char at 800or 1000 °C leads to a significant decrease in the resistance (Fig. 9), and the inductive impedance appears instead of the capacitance in the high-frequency range. This is due to a decrease in the content of surface functionalities and the formation of well-ordered carbon sheets. Calculations of real (*σ*′) and imaginary (*σ*′′) components of the complex conductivity (σ*) vs. frequency (*f*) show that the values of *σ*′ are greater by two to three orders of magnitude than that of *σ*′′ at *f* < 1 kHz. Estimation of the specific conductivity as the value of σ* at *f* → 0 (using the plot of σ* vs. log*f*) (Table 3) shows an increase in the conductivity with increasing temperature of char activation from 800 to 1000 °C because of the ordering of carbon sheets and a decrease in the content of O- and H-containing functionalities discussed above. Lactose-based carbons, which are most porous (Table 2), demonstrate a greater conductivity than other carbons (Table 3). This can be explained by a deeper treatment of the lactose-based carbons with removal of the O- and H-containing functionalities. Additionally, the DTA curve for lactose has the shape different from that for glucose and saccharose over a broad temperature range of 400–1000 °C (Fig. 1).

**Table 3:** Specific conductivity (Ω^−1^ m^−1^) of activated carbons

Precursor	Activation at 800 °C	Activation at 1000 °C
Glucose	6.3	32.2
Saccharose	12.5	61.2
Lactose	58.0	147.5

## Conclusions

Thermolysis of glucose, lactose, and saccharose in the inert atmosphere results in the mass loss up to 88% (saccharose), 84.3% (glucose), and 82.9% (lactose) during heating to 1000 °C. Chars prepared at 400 °C contain different O- and H-containing functionalities, whose amounts decrease during oxidizing activation at 800 and 1000 °C. However, according to the IR spectra, certain amounts of these functionalities remain even after heating at 1000 °C in the inert atmosphere. During heating of the chars in vacuum in the TPDMS experiments, desorption of O- and H-containing fragments is maximal at ~300 °C.

Oxidizing activation of the chars with controlled amounts of oxygen penetrating into the closed vessels through nanosized pores results in an increase in the porosity and specific surface area of carbons depending on the type of the used precursors. The maximum enhanced porosity is observed for lactose-based carbon activated at 1000 °C. For saccharose-based carbon, the difference in the porosity of samples activated at 800 and 1000 °C is relatively small. For glucose-based carbon, a very strong decrease in the porosity is observed for carbon activated at 1000 °C.

All carbons have nanopores and narrow mesopores; broad mesopores and macropores are practically absent. The hysteresis loops of the nitrogen adsorption-desorption isotherms are open for all chars and carbons with one exception of lactose-based carbon L1000 activated at 1000 °C and possessing the maximal values of *V*_p_ and *S*_BET_. These results can be explained by burn-off mainly of the outer layers of carbon particles and a small degree of burn-off in pores. The second process is maximal for L1000 as the most porous sample studied. An increase in the activation temperature leads to an increase in the conductivity of carbons that is maximal for L1000. This can be explained by deeper activation of this carbon and more effective removal of the O- and H-containing functionalities.

## Additional file

Additional file 1: Figures S1 and S2. Show electron diffraction patterns and pore size distributions of activated carbons. **Figure S1.** Electron diffraction patterns for samples (a) L800 and (b) S800. **Figure S2**. Pore size distributions calculated using the SCV/SCR method and TEM images treated using ImageJ with the granulometry plugin. (DOCX 400 kb)

## Abbreviations

2D-NLDFT: 2D-nonlocal density functional theory
AC: Activated carbon
BET: Brunauer-Emmett-Teller
DTA: Differential thermal analysis
DTG: Differential thermogravimetry
IR: Infrared
PSD: Pore size distribution
QSDFT: Quenched solid density functional theory
SCR: Self-consistent regularization
TEM: Transmission electron microscopy
TG: Thermogravimetry
TPDMS: Temperature-programmed desorption mass-spectroscopy
XRD: X-ray diffraction
