# Supplementary material for: Structural Features of Carbons Produced Using Glucose, Lactose, and Saccharose
Source: Nanoscale Res Lett. 2016 Nov 17;11:508. doi: 10.1186/s11671-016-1723-z (PMC5114214; doi:10.1186/s11671-016-1723-z)
Supplement: Additional file 1: Figures S1 and S2. — Show electron diffraction patterns and pore size distributions of activated carbons. Figure S1. Electron diffraction patterns for samples (a) L800 and (b) S800. Figure S2. Pore size distributions calculated using the SCV/SCR method and TEM images treated using ImageJ with the granulometry plugin. (DOCX 400 kb) [file 11671_2016_1723_MOESM1_ESM.docx]

Fig. S1 and S2 show electron diffraction patterns and pore size distributions of activated carbons


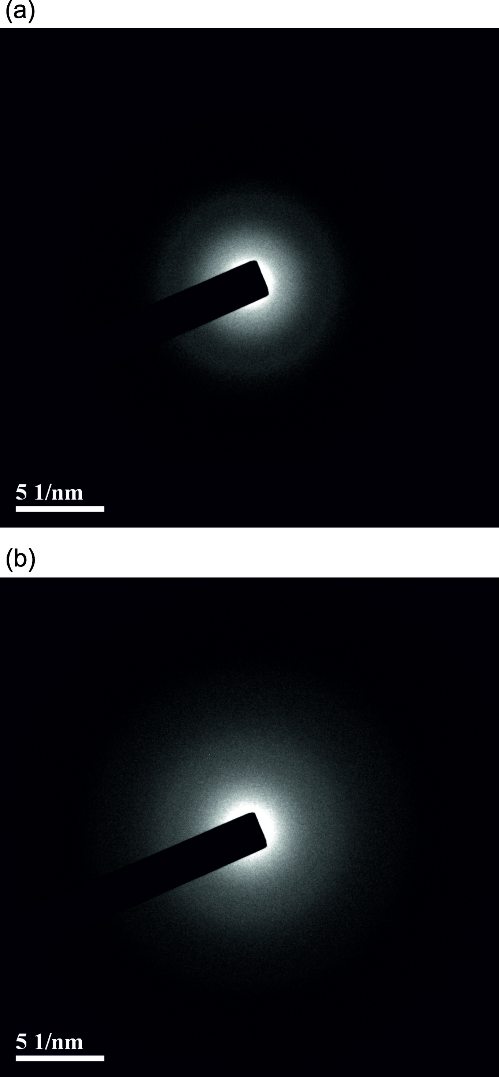


**Fig. S1.** Electron diffraction patterns for samples (a) L800 and (b) S800.





**Fig. S2.** Pore size distributions calculated using the SCV/SCR method and TEM images treated using ImageJ with the granulometry plugin.
